# The Structured Mind at Rest: Low-Frequency Oscillations Reflect Interactive Dynamics Between Spontaneous Brain Activity and a Common Architecture for Task Control

**DOI:** 10.3389/fnins.2022.832503

**Published:** 2022-07-11

**Authors:** Catherine Sibert, Holly Sue Hake, Andrea Stocco

**Affiliations:** Cognition and Cortical Dynamics Lab, Department of Psychology, University of Washington, Seattle, WA, United States

**Keywords:** brain architecture, cognitive architecture, computational models, Dynamic Causal Modeling, fMRI, resting state

## Abstract

The Common Model of Cognition (CMC) has been proposed as a high level framework through which functional neuroimaging data can be predicted and interpreted. Previous work has found the CMC is capable of predicting brain activity across a variety of tasks, but it has not been tested on resting state data. This paper adapts a previously used method for comparing theoretical models of brain structure, Dynamic Causal Modeling, for the task-free environment of resting state, and compares the CMC against six alternate architectural frameworks while also separately modeling spontaneous low-frequency oscillations. For a large sample of subjects from the Human Connectome Project, the CMC provides the best account of resting state brain activity, suggesting the presence of a general purpose structure of connections in the brain that drives activity when at rest and when performing directed task behavior. At the same time, spontaneous brain activity was found to be present and significant across all frequencies and in all regions. Together, these results suggest that, at rest, spontaneous low-frequency oscillations interact with the general cognitive architecture for task-based activity. The possible functional implications of these findings are discussed.

## 1. Introduction

Despite a shared goal of understanding the underlying mechanisms of the brain, research that focuses on high-level, large-scale structural models of cognition, such as cognitive architectures, remains largely isolated from efforts to interpret direct measurements of brain activity. Many neuroscientists are reluctant to rely on the results and conclusions from cognitive architectures because, while the behavior of the models often closely matches observed human data, the mechanisms driving that behavior are primarily rooted in computer science and information theory. Moreover, while efforts have been made to connect components of cognitive architectures to corresponding brain regions (Just and Varma, 2007; Anderson et al., 2008; Webb et al., 2013; O'Reilly et al., 2016; Samsonovich, 2020) direct, biological brain functions are rarely well-captured by the more conceptual architecture modules. In particular, these architectures may be making incompatible assumptions about the basic functional components needed to support cognition, and often struggle to capture the basic patterns of neuronal signaling. Thus, a challenge for these architectural network models is accounting for spontaneous, or resting state, brain activity, which occurs outside the constraints of task events that invoke activity in specific brain regions.

The spontaneous activity observed in the brain at rest exhibits many remarkable characteristics. It is oscillatory in nature (Fox et al., 2005) and concentrated in the low frequency band (<0.1 Hz). Because it is widespread and persistent, low frequency oscillations (LFOs) are believed to account for most of a neuron's energy expenditure, much more so than the localized bursts of activity that occur during task performance (Pezzulo et al., 2021). This burdensome metabolic cost suggests that LFOs must play a functionally important, if yet unknown, role in cognition. In addition, spontaneous activity at rest appears to be spatially and temporally organized. Specifically, LFOs are organized into different networks of regions, with regions within a network being more correlated than regions across networks (Fox et al., 2005; Power et al., 2011), and with rapid transitions that resemble task-related activity (Kang et al., 2019).

One major question on the nature of LFOs is the degree to which they are functionally related to a high level network architecture of the brain. As mentioned above, architectural models of cognition rely on the presence of some kind of underlying network system, but the structure and mechanisms are often only loosely tied to structures present in the brain. There is some evidence to suggest that a single high level network structure is supporting activity across tasks (Stocco et al., 2021; Rawls et al., 2022), and lower level analyses have found structural similarities in network activity across both states (Cole et al., 2014; Krienen et al., 2014; Bolt et al., 2017), but the degree to which a single, broad scope architecture is involved in the patterns of LFO activity at rest is still unclear.

Many hypotheses have been put forward to explain the functional nature of spontaneous LFOs, and most can be framed in terms of the relationship between localized, regional oscillations, and the larger network architecture of the brain.

The simplest explanation is that spontaneous LFOs are just noise from non-neural signals, such as changes in blood flow, that are introduced into the BOLD recording. According to this hypothesis, functional correlations between regions are merely a consequence of the synchronized noise driving them (Murphy et al., 2009; Wen and Liu, 2016; Das et al., 2021). However, once spontaneous LFOs are properly modeled and accounted for at the regional levels, the contributions of intrinsic connectivity between regions is null.

A second group of hypotheses assumes that spontaneous brain activity aims to re-process events and maintain generic priors (Raichle and Snyder, 2007). According to this family of hypotheses, regions spontaneously rehearse patterns of activity they have previously learned in order to maintain connections and prevent catastrophic interference. In this view, functional correlations between different areas would reflect the probability of co-occurrence of these events, due to one pattern of activity in one region spontaneously spreading over to a different region. Therefore, once regional fluctuations of activity are modeled, there might still be residual intrinsic connectivity between regions, but such residual connectivity would not necessarily resemble the overall brain architecture needed for goal-oriented, task-based activity.

A third group of hypotheses suggests that spontaneous brain activity is a different cognitive state. That is, spontaneous brain activity could simply be “spontaneous thought,” such as the natural occurrence of mind-wandering events that intrude during task-based activity and likely dominates the idle periods of rest (e.g., Gruberger et al., 2011; Vago and Zeidan, 2016). In this view, resting-state activity is mostly top-down processing that idles while waiting for stimuli, and the intrinsic connectivity network that drives task-based activity will dominate the patterns of low-frequency oscillations during any period of directed activity. If this is the case, then once it is modeled, few (if any) regional sources of spontaneous brain activity should remain.

Finally, a fourth group of hypotheses suggests that both local events and the intrinsic task-based activity are jointly necessary to explain the complex patterns of spontaneous brain activity at rest. For example, Pezzulo et al. (2021) recently proposed that spontaneous brain activity is used to refine the brain's predictive models during rest; as such, it requires both local sources that generate plausible inputs (which account for the region-specific nature of LFOs) and the general architecture for task-based connectivity (which is used to make generative predictions and is refined during resting-state activity).

These explanations can be distinguished on the basis of the relationship between spontaneous oscillations and task-based activity and, specifically, by separately modeling LFOs and network connectivity during rest. If this were possible, the first, “noise” hypothesis would entail that intrinsic oscillatory dynamics are sufficient to explain region patterns of activity without the need to include network connectivity. Furthermore, the weights and phases of different oscillators should be highly correlated because they would likely reflect underlying physiological signals. The second hypothesis (i.e., the “priors”) also entails that network connectivity is either not needed or does not resemble the network structure of task-based activity, since all the regional variance in activity could be explained by different regions being synchronized over different rhythms. The weights of different frequencies and phases, however, would not be similar across networks, and the different correlations between regions belonging to different networks would be shown by different weights of regressors modeling different frequencies and phases.

The third hypothesis (i.e., the “alternative cognitive state”) suggests that activity during rest and tasks are separate states of cognition, each with a structured, but distinct pattern of network connectivity. The fourth hypothesis (i.e., the “predictive model”) also requires a notion of underlying network connectivity during both task activity and rest, but here the LFO activity serves to update and refine the network structure used during a task, and the network structure should be the same for both states, along with similar patterns of activity.

One obstacle to disentangling these hypotheses is the inherent ambiguity in the term “network connectivity observed at task.” As noted by Pezzulo, task-based activity tends to be different based on the specific requirements of the task. Thus, to properly answer this question, one needs a valid model of what a general “task-based” network of regions is.

**Figure 1:**
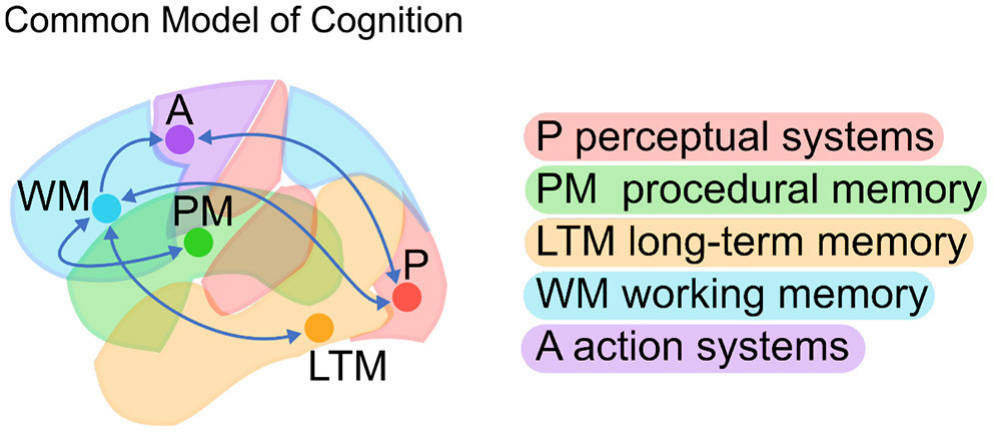
The Common Model of Cognition (CMC; Adapted from Laird et al., 2017).

A possible answer comes in the form of the Common Model of Cognition (CMC); a high-level, large-scale consensus model drawn from decades of research in cognitive architectures (Laird et al., 2017). The CMC is a computational framework that can serve as a blueprint to understand the organization of a human-like mind. Abstract computations are categorized into five functional components (perceptual systems, procedural memory, long-term memory, working memory, and action systems) with specific directional relationships between them (Figure 1).

Although it was not proposed specifically as a brain architecture, a number of studies have found that the CMC is surprisingly effective at modeling brain activity across tasks and individuals (Steine-Hanson et al., 2018; Stocco et al., 2021). In this interpretation, the CMC's functional components are mapped onto large-scale brain regions and their relations are translated into predicted patterns of functional connectivity. In other words, the neural counterparts of the functional components and their connections serve as a simplified architecture for the human brain. Furthermore, the same broad architecture has been shown to successfully capture the large-scale organization of brain activity across a wide range of cognitive domains, such as response inhibition, problem solving (Steine-Hanson et al., 2018), working memory, emotion recognition, decision-making, analogical reasoning, language, mathematical cognition, and social inference (Stocco et al., 2021). In fact, these studies found that not only did the CMC capture brain activity across tasks and domains but that it provided a comparatively better fit than multiple alternate architectures derived from the cognitive neuroscience literature.

Because of its generality across tasks, the CMC offers a unique opportunity to understand the functional dynamics of LFOs in the resting brain and their relationship to the intrinsic network activity that governs cognition in the task-state. To this aim, this paper extends the work of Stocco et al. (2021) by testing the CMC on brain activity at rest using a pre-defined network of brain regions. Specifically, this paper compares the CMC against six other exemplar network structures in an effort to capture the underlying structure of the mind at rest.

Using the CMC as a framework through which to predict resting state activity can provide a high-level context in which to interpret the role of LFOs in the resting state. First, it can help examine the question of whether or not a network structure is present at all in the resting state. If not, all candidate models of network structure, including the CMC, should provide equally sufficient, or more likely, equally poor, accounts of brain activity during rest. If this is the case, examining the weights assigned to the input oscillators could distinguish between the noise hypothesis (where the weights and phases should be highly correlated to reflect the external driving forces) and the generic priors hypothesis (where weights and phases should be distinct). Alternatively, if a candidate model structure does provide a significantly better account of activity than the others, this would suggest the presence of an underlying network structure driving LFO activity. If the alternate state hypothesis is true, and resting state is a distinct cognitive mode, the best network structure should not be the same network that provided the best account of task-based activity, the CMC (Stocco et al., 2021). However, if the predictive model hypothesis is correct, and LFO activity serves to refine a single network structure, then the CMC should provide the best account of brain activity in resting state as well as in tasks.

## 2. Materials and Methods

### 2.1. Dynamic Causal Modeling

To separate the effects of intrinsic network connectivity between regions from spontaneous oscillations, regional brain activity was modeled using Dynamic Causal Modeling (DCM; Friston et al., 2003). DCM is a model-based technique used to estimate, fit, and compare hypothetical network models to fMRI data. Because it is a top-down, hypothesis-driven method that provides directional estimates of connectivity, it is particularly suited for this investigation (Stocco et al., 2021). Furthermore, DCM offers additional robustness in the face of regional differences because it explicitly estimates the parameters of the hemodynamic response function for each region (Friston et al., 2013). The DCM framework is a point-mass neural modeling approach in which changes in the activity in a set of brain regions is modeled through a linear combination of the effects of other regions and external factors:


(1) $$\begin{array}{l} dy/dt=\text{A}\text{y}+\text{C}\text{x} \end{array}$$

In this equation, hemodynamic brain activity, represented by vector **y**, is multiplied by matrix **A**, which contains a set of parameters capturing the proposed directional connectivity between regions. Thus, the structure of matrix **A** can be adapted to test alternative connectivity architectures. **C** is a matrix of the parameters that specify how external or driving inputs elicit changes in brain activity, and **x** defines the design matrix of task inputs. In typical, task-based analyses, the **C** matrix contains the onsets and durations of external events corresponding to different task conditions. For task-free resting state data, where there are no external inputs driving activity, the **C** matrix was adapted to model low frequency fluctuations at different frequencies and phases.

### 2.2. Alternate Model Architectures

As pointed out in Stocco et al. (2021), DCM is a strictly top-down, theory-driven method, and cannot be used to infer an architecture from the data. Instead, to evaluate the CMC as an architecture, its predictions were compared against a collection of alternative networks that consist of the same components, but different connection patterns (Stocco et al., 2021). These alternate models are not exact implementations of other cognitive architecture systems, like ACT-R or SPAUN, but instead represent the space of possible theoretical neural architectures.

**Figure 2:**
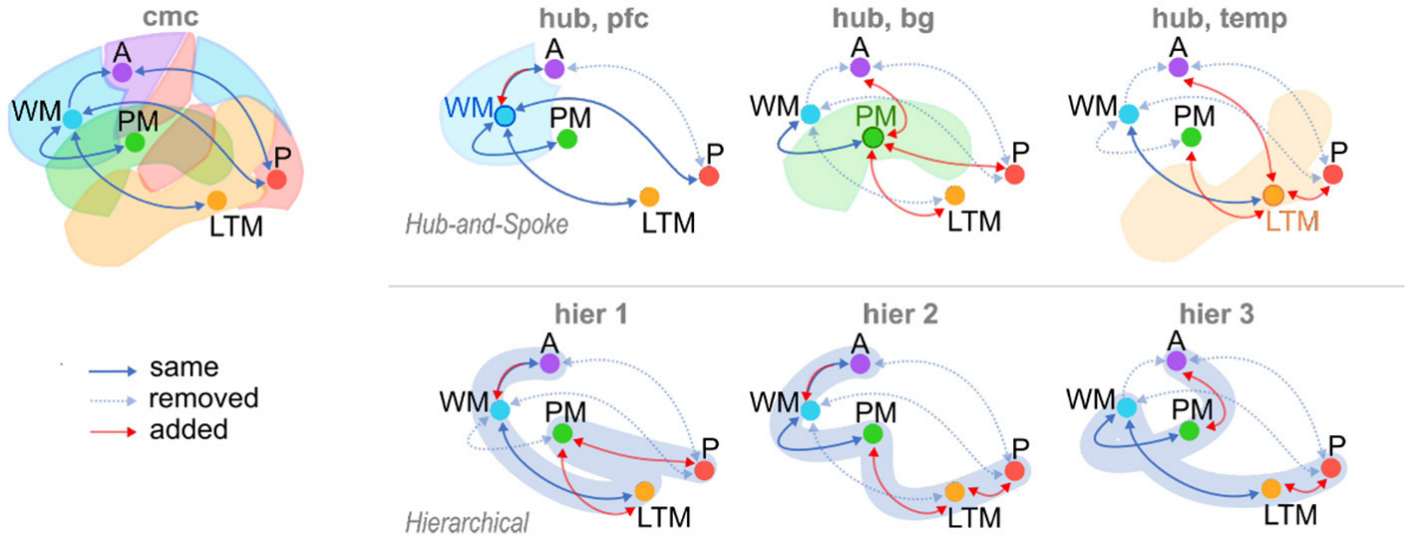
The Common Model of Cognition (CMC) and six alternate model architectures. Alternate models consist of three variations of Hub-and-Spoke (hub) models, and three variations of Hierarchical (hier) models. Arrows: “same,” connections present in both CMC and candidate models (solid blue); “removed,” connections present in CMC and removed in candidate models (dotted blue); “added,” connections added to candidate models and absent in CMC (red).

The alternate architectures fall into two broad categories, or families. In the “Hub-and-Spoke” family (Figure 2), a single region of interest (ROI) is designated as the central “Hub,” and is bidirectionally connected to all other ROIs. However, none of the “Spoke” ROIs are connected to any other—all activity must travel through the “Hub.” Three different Hub-and-Spoke models are considered, based on whether the role of the hub is played by the Prefrontal Cortex, mapped to Working Memory (as proposed by Cole et al., 2012), the basal ganglia, mapped to Procedural Memory (as proposed by Anderson, 2007), or the temporal lobe, mapped to Long Term Memory (as proposed by Visser et al., 2010). The “Hierarchical” family of models proposes an alternate structure, wherein brain connectivity implements hierarchical levels of processing that initiate with Perception and culminate with Action (Figure 2). Networks in this family conceptualize the brain as a feedforward neural network model in which different regions perform progressively greater levels of representational abstraction (Huntenburg et al., 2018). Three different hierarchical architectures are generated based on the relative position of the basal ganglia (mapped to Procedural Memory) in the hierarchy. Specifically, the basal ganglia can be placed between perception and long-term memory (as in models of procedural categorization: Seger, 2008; Kotz et al., 2009), between long-term memory and working memory (as in models of memory retrieval: Scimeca and Badre, 2012), or between working memory and action (as in models of action selection: Houk et al., 2007).

Broadly speaking, the CMC can be considered as a “Hub-and-Spoke” structure, using Working Memory (mapped to the Prefrontal Cortex) as the “Hub” ROI, with an additional direct connection between Perception and Action.

### 2.3. The Human Connectome Project Dataset

The data used in this analysis was drawn from the Human Connectome Project (HCP), a large-scale effort to collect neuroimaging data from healthy young adults. This study in particular analyzed a subset (*N* = 168) of rsfMRI data exclusively. For each subject, 14 min of rest data (eyes open with fixation) were recorded prior to a run of task data collection. A second rest run was recorded after the task battery, and was not included in this analysis. Between the two collection days, each subject had a total of 28 min of data. Each day's data was modeled separately, and then combined in the final analysis.

### 2.4. Data Processing and Analysis

#### 2.4.1. Image Acquisition and Preprocessing

MRI images were acquired and minimally preprocessed according to HCP guidelines (Poldrack et al., 2011; Barch et al., 2013; Essen et al., 2013). Scans were taken on a 3T Siemens Skyra using a 32-channel head coil with acquisition parameters set at TR = 720 ms, TE = 33.1 ms, FA = 52°, FOV = 208 × 180 mm. Each image contained 72 2.0 mm oblique slices with an in-plane 2.0 × 2.0 mm resolution. Images were acquired with a multi-band acceleration factor of 8X. These raw images then underwent minimal preprocessing including unwarping, motion realignment, and normalization to the standard MNI template. Motion artifacts were removed through linear regression, using a model that included the three axial (over x, y, and z dimensions) and the three rotational (pitch, yaw, and roll) volume-by-volume movement estimates as well as their first-order derivatives (Ciric et al., 2017). The images were then smoothed with an isotropic 8.0 mm full-width half maximum Gaussian kernel, which is the same amount of smoothing used in Stocco et al. (2021).

#### 2.4.2. Low-Frequency Oscillations

**Figure 3:**
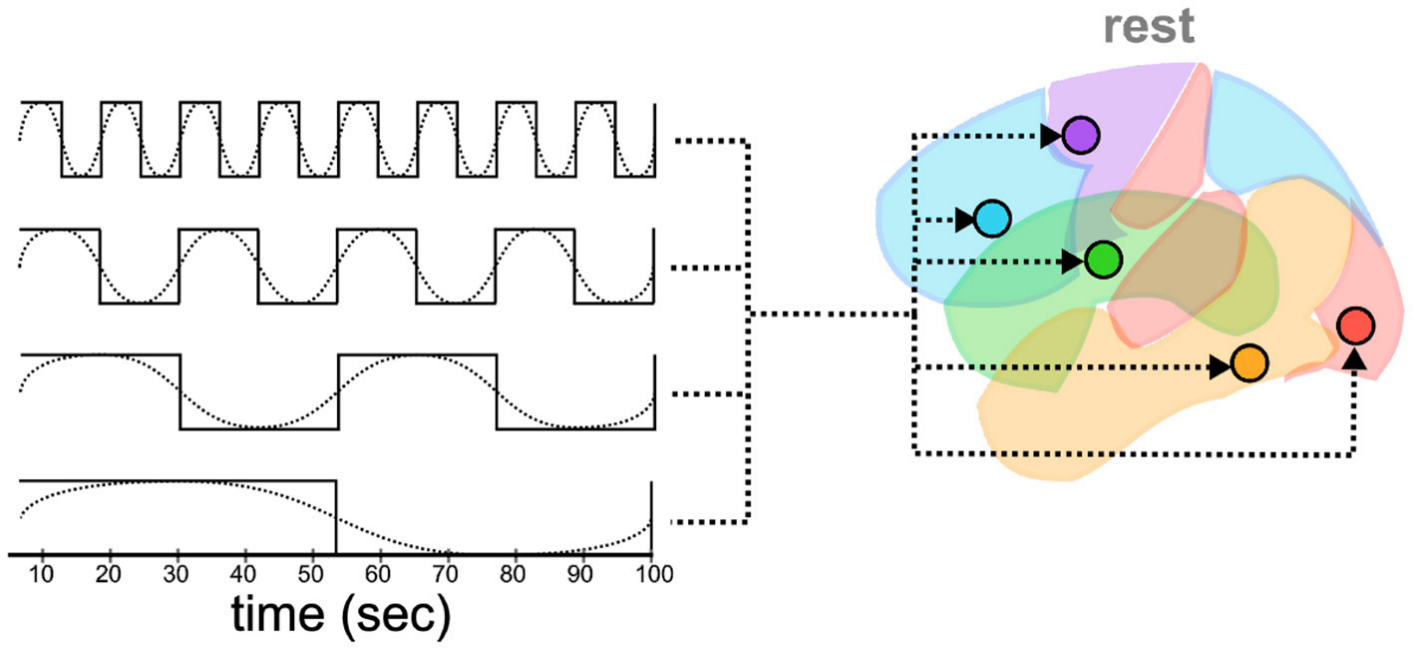
Oscillatory waves of different frequencies (dotted lines) are and phases (not shown) translated into binarized “box-car” plots of events (solid lines). Each event is then simulated as a driver of activity in all ROIs.

Both general linear modeling (GLM) and DCM analysis require a design matrix that specifies the timing of external events that drive brain activity. Traditionally, these events are task-related and describe the onset and duration of some experimental stimuli. Rest data, by contrast, is collected without any specific task structure, and the recorded activity must be driven by internal and unobservable patterns. Following Di and Biswal's (2014) method, a series of slow oscillatory waves of different frequencies were created as input “events” that simulate background brain activity (Figure 3). Specifically, eight different driving waves were generated as sine and cosine waves with frequencies of 0.01, 0.02, 0.04, and 0.08 Hz, respectively. The frequencies of these oscillations capture the canonical frequency range (0.1–0.01 Hz) of spontaneous low-frequency fluctuations in brain activity (Fox et al., 2005). Because DCM only allows binary regressors, each wave was subsequently binarized, with all the values in the positive cycle of the wave set to 1 and the others set to 0.

Thus defined, these regressors capture a large range of possible oscillatory effects across frequencies and phases, covering the entire timeline of the experiment even after being discretized (Figure 3). Note that, because DCM analysis does not rely on the partition of variance across regressors, the simulated oscillatory events do not need to be orthogonal (Friston et al., 2003; Murphy et al., 2009), in fact, in the most noteworthy cases, they are not; they just need to adequately capture the hypothetical drivers of neural activity.

An important assumption in a DCM analysis concerns how different simulated events affect the different regions. In task-based DCM analysis, it is possible to make reasonable assumptions about which regions are affected by which events based on functional specialization. For example, one might reasonably assume that the presentation of visual stimuli would directly affect only the perceptual region, and affect other regions only downstream and indirectly. In resting-state fMRI, however, the functional role of each region cannot be used as a guideline for associating events to ROIs, and a different strategy must be found. In their previous work, Di and Biswal's (2014) explored a subset of possible regressor-by-region combinations to determine the most appropriate. Here, we followed the procedure of Wapstra et al. (2022) and let each region be potentially affected by each oscillatory regressor (Figure 3). We chose this approach for two reasons. First, it is the most general, and permits us to directly examine whether each region's activity includes fluctuations that cannot be fully explained by the effects of other regions. Second, this approach goes *against* our hypotheses that spontaneous brain activity would follow a structured architecture, as it gives every region the greatest opportunity to have its time series modeled by external inputs rather than by the network effects of other regions.

#### 2.4.3. Regions of Interest Definition

Previous DCM analyses relied on task-based activity to define specific regions for each model component, but in the absence of a task structure for rest data, an alternate method was needed to determine regions based on prior assumptions. One possibility could be to use large-scale networks derived from functional parcellations of brain anatomy, such as those proposed by Power et al. (2011) and Yeo et al. (2011). Indeed, and as noted in Stocco et al. (2021), both methods identify a number of networks that is roughly on par with the components of the CMC, with some of them having straightforward translations within the CMC's functional components (e.g., the sensorimotor network in Yeo and the motor component in the CMC). The use of these networks, however, has some drawbacks. The first is that, although some networks can be associated with functional components, such associations are necessarily *post-hoc* labels and might not cover exactly the scope of the corresponding function. For example, although the Long-Term Memory component should certainly encompass the medial temporal lobe (Stocco et al., 2021), should it extend to the Default Mode Network? And should the Working Memory component be associated with the Frontoparietal Network, the Dorsal and Attention networks, or both? A second problem is that, being derived from resting-state functional connectivity itself, the use of a priori parcellated networks in this analysis runs the risk of “double-dipping” (Kriegeskorte et al., 2009): a network architecture derived from resting state activity would naturally be better off explaining resting state activity.

**Figure 4:**
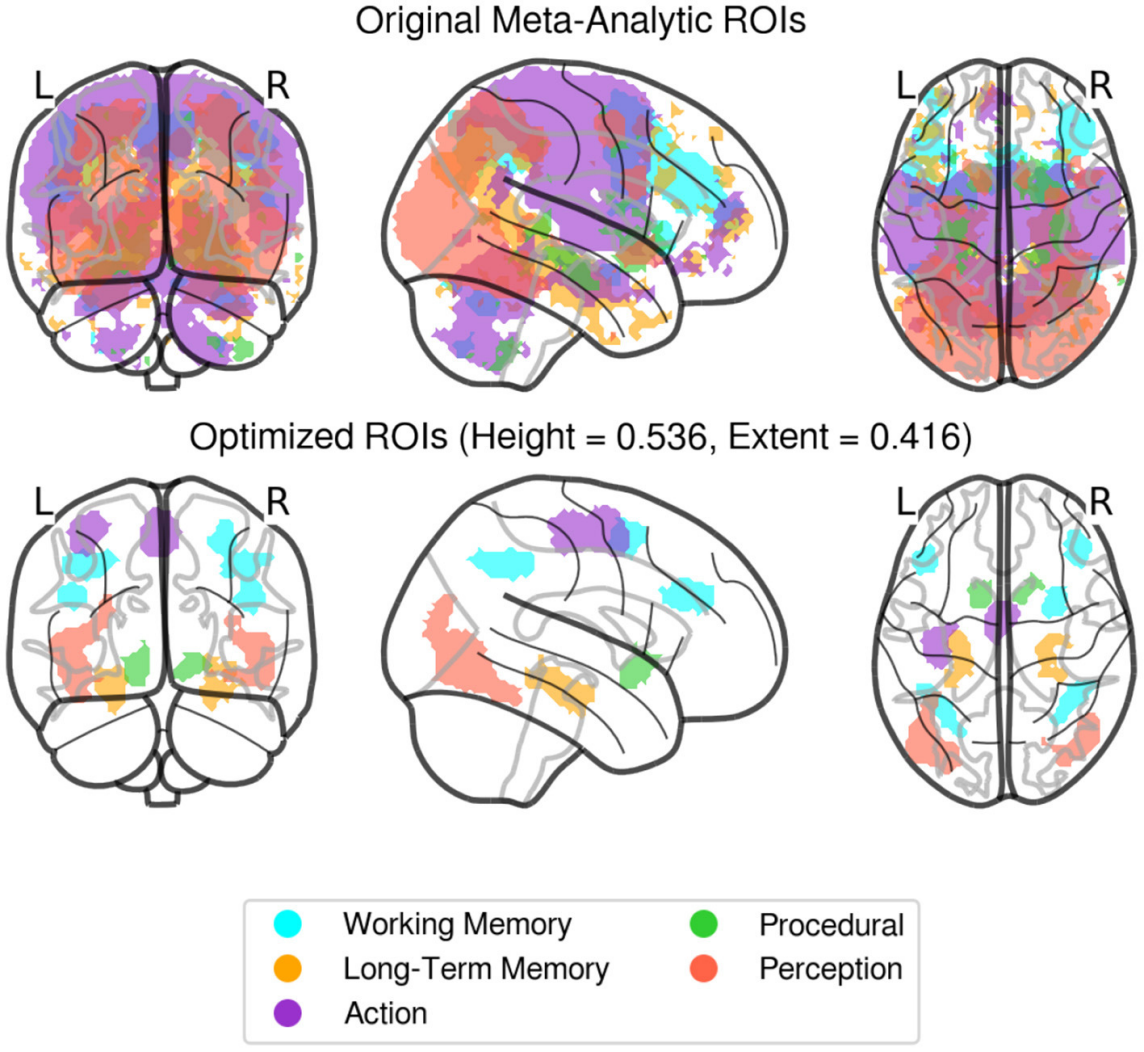
Original (non-thresholded, **top**), and Optimized (final, **bottom**) regions of interest derived from Neurosynth activity masks. Individualized ROIs were created by identifying voxels responding significantly (*p* < 0.05) to any of the waveform regressors, within these predefined areas for each subject.

Instead, in this paper we took the more agnostic approach of identifying large-scale ROIs from a priori, top-down functional associations derived from meta-analysis of task-based neural activity. Specifically, initial region masks were created using NeuroSynth (www.neurosynth.org), a platform that combines the results of thousands of published fMRI results and produces meta-analysis images of activity associated with various higher level conceptual category terms. For each of the five components of the CMC model, a corresponding term was chosen from NeuroSynth's database, and a summary statistical mask was produced for each term, with each voxel having an associated *Z*-value representing the probability that the voxel would show up in a study associated with the term. The following terms were searched in Neurosynth: Perception = “Visual,” Action = “Motor,” Working Memory = “WM,” Procedural Memory = “Learning,” Long Term Memory = “LTM.” These individual masks, however, were large and produced significant overlap when combined, meaning that activity in a particular voxel could belong to more than one region. To solve this problem, two thresholds were applied to the original masks, one height threshold applied to each individual voxel statistic and a minimal extent threshold applied to each cluster size. Both thresholds were calculated proportionally for each region, i.e., as a proportion of the highest Z-score and of the largest cluster within an image, respectively. The proportional adjustment was done to prevent regions with large clusters and high statistics, like perception, from overtaking regions with comparatively low Z score levels, like procedural memory. The Nelder and Mead (1965) optimization algorithm was then applied to find thresholds in the two-parameter space that would produce the largest possible regions without any overlapping voxels. The final values identified by the Nelder-Mead algorithm were a proportional height threshold *H* = 0.5359, and a proportional extent threshold *E* = 0.4164. The final ROIs masks are shown in Figure 4. To account for residual differences in individual functional anatomy, individualized ROIs were created by applying the Neurosynth-derived mask to all the voxels that showed a significant (*p* < 0.05) response to any of the oscillatory regressors. These voxels were identified with an *F*-test, using the procedure described in Section 3.

Finally, for each subject and individualized ROI, a representative time-series was created by extracting the eigenvariate (i.e., first principal component) of the time-series of all voxels within that mask. The use of the first principal component or the *eigenvariate*, as opposed to other dimensionality-reduction methods (such as simple average across voxels) is recommended and is standard practice in the DCM literature (Friston et al., 2003). It is also particularly important in this context, since the first principal component is a more robust estimator in the presence of spurious voxels that behave significantly different from the others within the ROI. We reasoned that such spurious voxels were significantly likely to occur in our analysis because our regions had been created from a meta-analysis of various functional studies and were defined at the group level, with only minor adjustments to the individual functional anatomy.

#### 2.4.4. Model Fitting

For each subject in each resting state session, a time-series signal was extracted from each of the five regions defined in the previous section. These regions were connected according to the specifications of each model structure (Figure 2), creating seven possible accounts of network structure. Using the oscillatory signals as input, each model generated a predicted time course of the BOLD signal by applying a biologically-plausible model of neurovascular coupling to the simulated neural activity of each region. Activity in each region was affected by oscillatory inputs as well as the propagation of activity through the network of connections specified by each model, and modified by parameters representing the strength of each connection, as encoded in the **A** and **C** matrix terms of Equation (1). An expectation-maximization procedure (Friston et al., 2003) was then iteratively applied to modify the parameters and reduce the difference between the predicted time course of the BOLD signal in each ROI produced by the model simulation and the actual BOLD time course extracted from the subject.

#### 2.4.5. Model Comparison

The models were compared on the basis of their likelihood function. The likelihood of a model *m* given data *x*, denoted as *L*(*m*|*x*), is formally expressed as the probability of it producing the observed data *x*; that is, *L*(*m*|*x*) = *P*(*x*|*m*). Assuming that participants are independent, the group-level likelihood values for a model *m* can then be expressed as the product of the likelihood of that model fitting each participant p, i.e., ∏_*p*_*L*(*m*|*x*_*p*_). The log-likelihood is the sum of all of the individual log-likelihoods: $\underset{p}{\sum }\log L\left(m|x_{p}\right)$. Although more sophisticated model comparison procedures have been proposed (e.g., Stephan et al., 2009), the log-likelihood based metric used here is not only the most easily interpretable, but also the most relevant, as it specifically applies to cases in which it is assumed that the model is constant or architectural across individuals (Kasess et al., 2010).

## 3. Results

### 3.1. Regressor Quality Analysis

**Figure 5:**
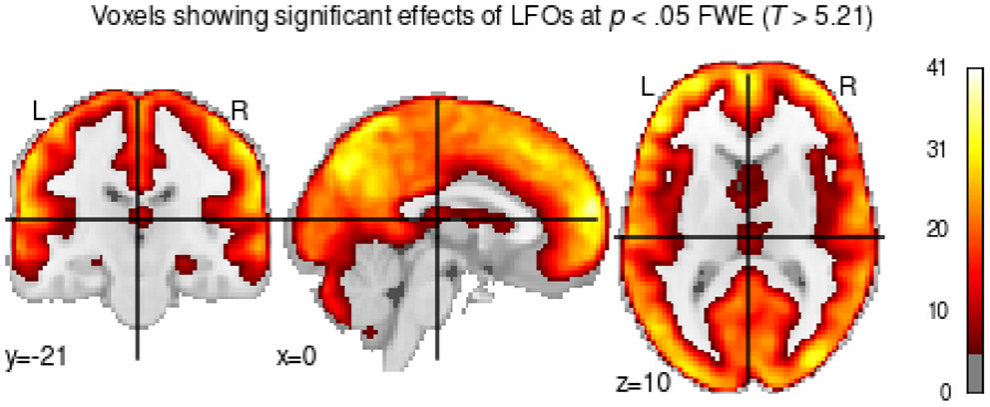
*T*-test showing voxels whose brain activity was significantly captured by the oscillatory regressors.

Before proceeding with our analysis of the DCM data, we first conducted a GLM analysis to ensure that our oscillatory regressors successfully captured brain activity. This is important because our analysis is predicated on the assumption that our simulated oscillatory regressors do successfully capture the patterns of spontaneous brain activity at rest, despite their highly simplified nature (Figure 3). To ascertain that this was the case, we calculated an omnibus ANOVA across all oscillatory regressors at the participant level. This test captures any variance that can be accounted for by any of the oscillatory regressors. The resulting F-statistic map was then log-transformed, yielding a measure of the difference between the variance explained by regressors and the residual variance (i.e., noise). Finally, a group-level *T*-test was performed on the individual-specific log-transformed F-maps. The result of this analysis is a statistical test of whether the variance captured by the regressors was significantly greater than the variance of the residuals. The results are shown in Figure 5, thresholded at a value of *t*_(160)_>5.212, which corresponds to *p* < 0.05 when corrected for multiple comparisons through the conservative Family Wise Error correction procedure.

As Figure 5 shows, most of the gray matter voxels exhibit oscillatory activity that was captured by our regressors. Importantly, the significant voxels encompass regions in all of our predefined ROIs, including the medial temporal lobes (long-term memory ROI in Figure 4) and the subcortical basal ganglia (procedural memory ROI in Figure 4), which are notoriously affected by lower signal-to-noise ratios in high-density neuroimaging protocols.

### 3.2. New ROI Analysis

**Figure 6:**
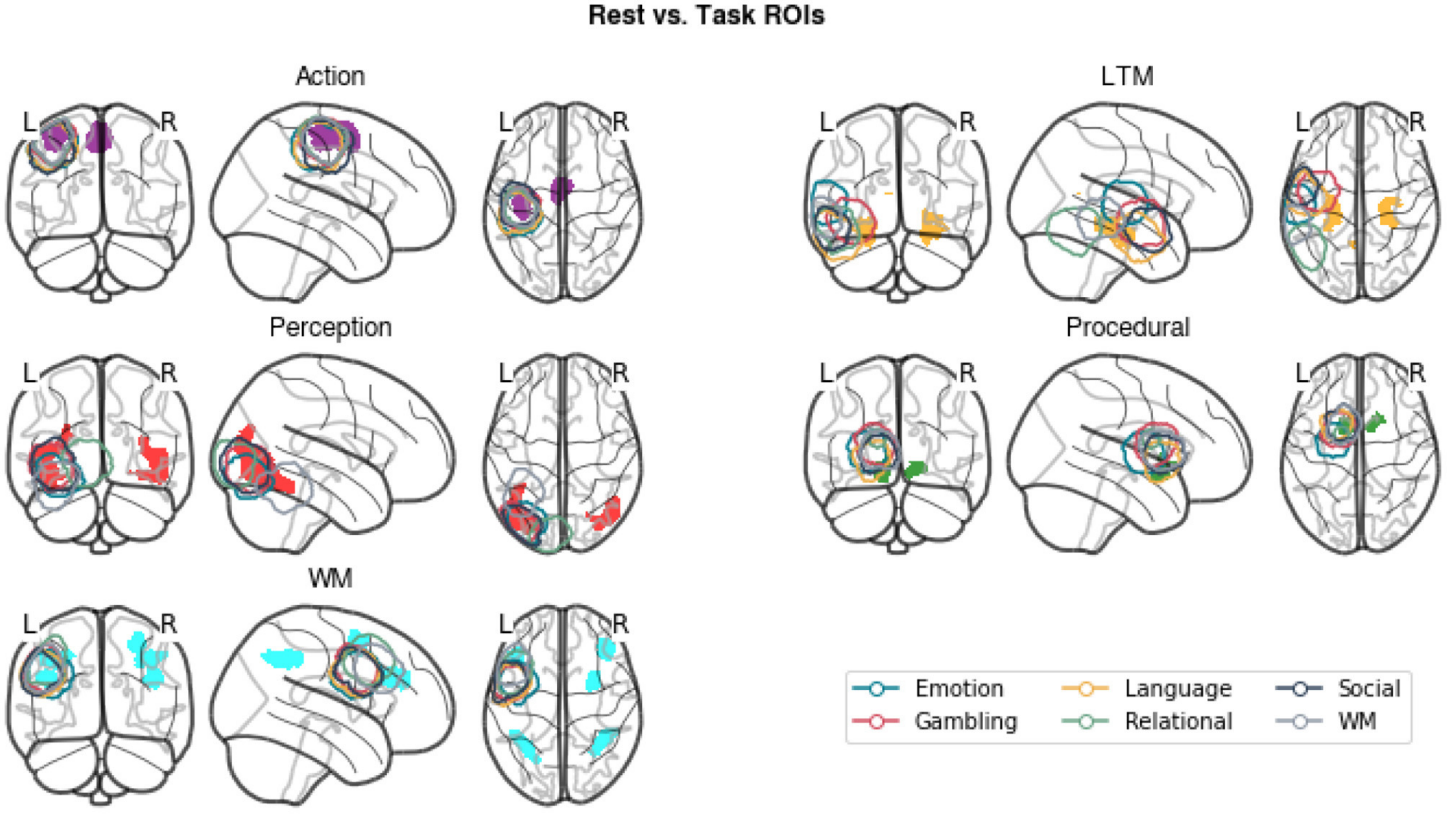
A comparison of the meta-analytic resting-state ROIs (gray) and the group-inclusive task-based ROIs (colored contours) from Stocco et al. (2021).

In addition to examining the amount of variance explained by our regressors, we conducted a second analysis to examine the extent to which the newly generated ROIs were comparable with the task-derived regions. This was done to ensure that our results remained compatible with the findings of Stocco et al. (2021) despite the different methods to identify the regions of interest. To this aim, we computed group-level ROIs from the original data from Stocco et al. (2021). For each task and ROI, a group-level inclusion region was created by first creating a binary mask of the corresponding ROI in each participant and then summing up all of the resulting masks. The resulting ROIs include all voxels that were present in at least one individual for that task. Figure 6 shows that the newly generated resting state ROIs are highly overlapping with the task-based ROIs. In the majority of cases, all task-specific ROIs overlap at least partially with the meta analysis-derived ROIs, with the overlap over sensorimotor regions (Perception and Action components) being the largest. The only exceptions are associated with LTM and WM regions. The LTM regions are strictly localized in the hippocampus for the resting-state but more broadly distributed to other medial temporal lobe regions for the task-based ROIs. This is likely due to a bias inherent in the localization method used for task-based fMRI (Stocco et al., 2021), which focuses on peaks of activation and penalizes regions with low signal-to-noise ratios, such as the hippocampus (Stark and Squire, 2001, < 0). The WM task-based regions are centered over the left dorsal prefrontal region that corresponds to an homologous right-hemisphere cluster for the resting-state WM region. This is likely due to the original choice made by Stocco et al. (2021) to include only left-lateralized ROIs in their analyses. In addition, the resting-state WM region includes other dorsal and caudal prefrontal clusters that were not included in the original study, but more closely correspond to the fronto-parietal network that supports working memory function (Stocco et al., 2021). Overall, the high degree of overlap between the task-based ROIs and our meta-analytic ROIs suggests that our results are comparable to those of Stocco et al. (2021).

### 3.3. Comparison of Architectures

Having ascertained the validity of the oscillatory regressors and of the similarity of the neurosynth-derived ROIs with the previous task-based results, we proceeded to examine the relative fit of different functional brain architectures to the resting-state data. Given that the different architectures contain different numbers of parameters, it is possible that higher relative log-likelihood is simply due to higher degrees of freedom. Because log-likelihood is not sensitive to model complexity, it is common to compute log- likelihood in some penalized form. For example, the common Akaike Information Criterion (AIC) and Bayesian Information Criterion (BIC) penalize likelihood by the number of parameters. Both measures assume, however, that parameter values are independently distributed, which is not the case for DCM models (for example, connectivity values for the same node tend to be correlated). For this reason, as in agreement with the DCM literature, we used a different, penalized form of likelihood known as Free Energy (Penny, 2012), which accounts for non-independent parameters.

**Figure 7:**
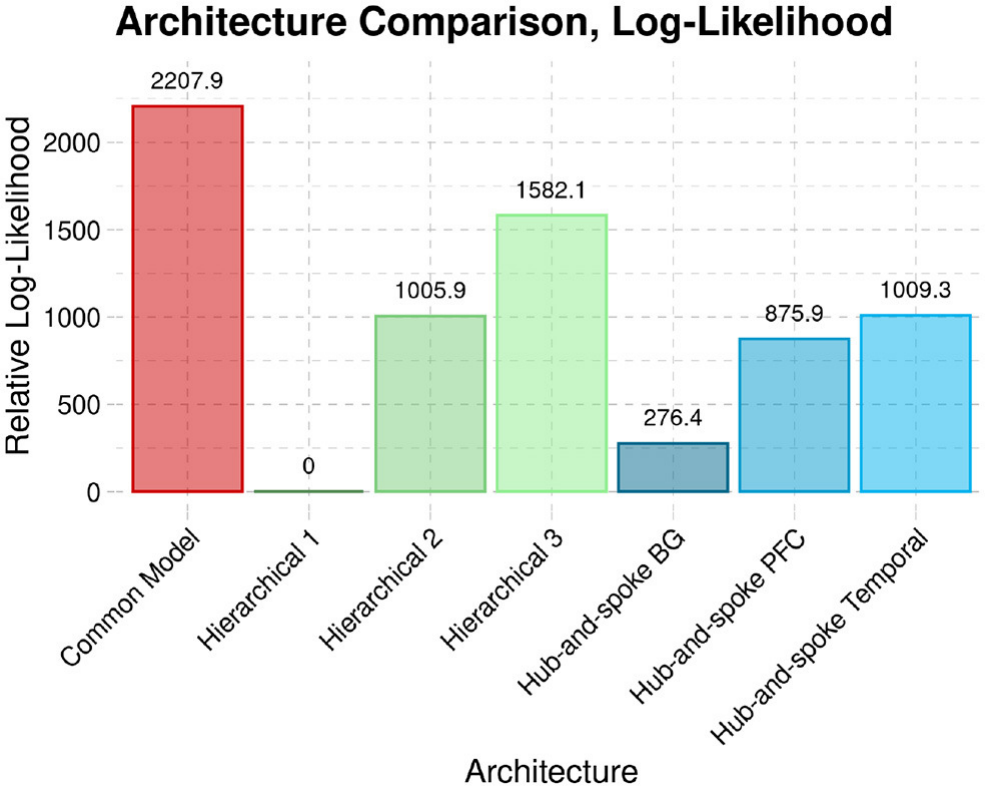
The penalized log-likelihood (Free energy) of the CMC architecture compared to six alternate architectures across both sessions of rsfMRI data.

For the comparison analysis, each session was modeled individually, and then both sessions were combined on a subject-level basis. Figure 7 illustrates the group-level penalized log-likelihoods of the different architectures in the rest condition. Note that the figure presents relative log-likelihoods: the lowest log-likelihood is subtracted from all the others. As a result, the worst-fitting architecture always has a relative log-likelihood value of zero, with the best-fitting architecture having the highest positive value.

Across both sessions, the CMC provides the best account of resting state brain activity when compared against each of the six alternate structures.

### 3.4. Analysis of Bayes Factors

Although the evidence in favor of the CMC is apparent, one might wonder exactly how significant the difference in log-likelihood is. To express log-likelihood in an interpretable form, we will use Bayes Factors (BF). The *BF*_1,2_ between two models *m*_1_ and *m*_2_ is defined as:


$$BF_{1,2}=P\left(m_{1}|\text{x}\right)/P\left(m_{2}|\text{x}\right)$$

In other words, the value of *BF*_1,2_ represents the odds of model 1 fitting the data better than model 2. Given the definition of likelihood as *L*(*m*|*x*) = *P*(*x*|*m*), *BF*_1,2_ can be expressed as:


$$BF_{1,2}=e^{\Delta L}$$

where Δ*L* = log*L*(*m*_1_|*x*) − log*L*(*m*_2_|*x*) is the difference in log-likelihoods between model 1 and model 2. As a guideline, Kass and Raftery (1995) suggest that values of BF > 20 correspond to a value of *p* < 0.05 in a canonical null-hypothesis test and provide “strong” evidence in favor of model 1 over model 2, while values of BF > 150 provide “very strong” evidence. All of the BF values for the comparisons of the CMC against all the other models exceeded 10^250^, indicating that the evidence in favor of the CMC is, in fact, overwhelming.

### 3.5. Random-Effects Analysis

Although the results provide strong evidence in favor of the CMC, it should be noted that they are not directly comparable with the model comparison approach reported by Stocco et al. (2021). In the original paper, the authors compared the different architectures by measuring the relative probabilities that each architecture would fit any given participant (Stephan et al., 2009). This approach is conceptually different from the log-likelihood approach because it is based on relative, rather than absolute, fit to the data and because participants are considered as a random factor, thus giving different architectures the opportunity to fit different subgroups of participants.

**Figure 8:**
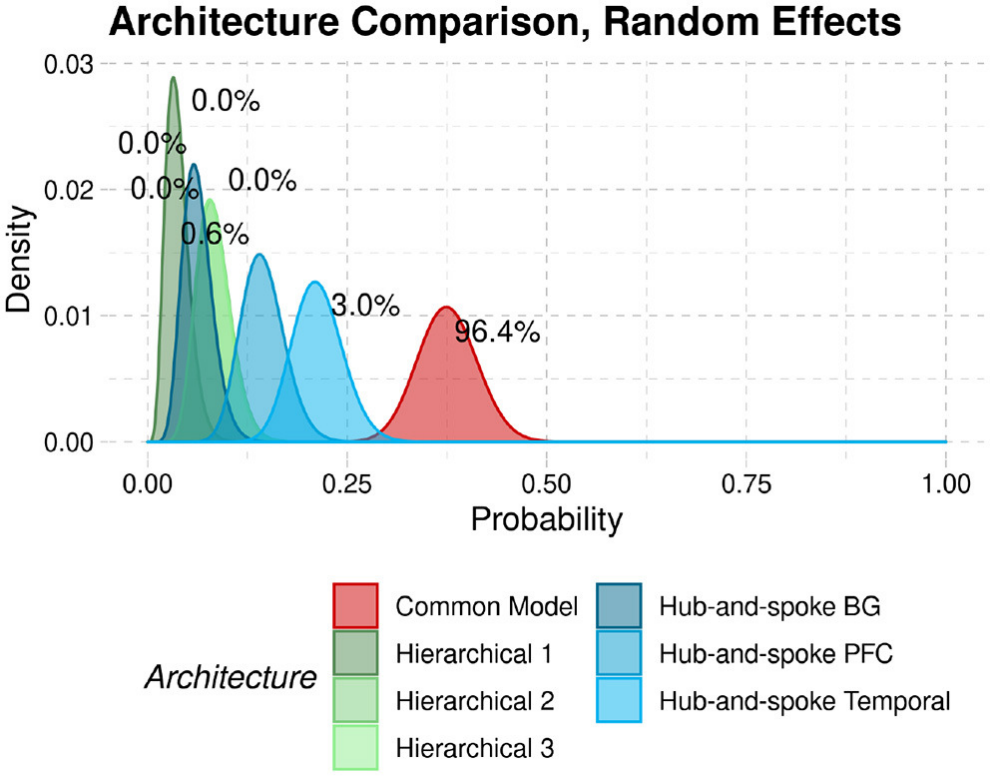
Probability densities that each architecture would best fit the data from a participant in our sample. Numeric labels represent the exceedance probabilities associated with each distribution.

To provide a better comparison to the original findings, we replicate the analysis method of Stocco et al. (2021) with the current resting-state data. The results are reported in Figure 8. In the figure, the curves represent the densities of the relative probabilities that each architecture would fit a participant. The superiority of the CMC is shown by the fact that its probability density function lies to the right of all other architectures. Architectures can be quantitatively compared in terms of exceedance probabilities, i.e., the probability that a point randomly sampled from their density distributions would have a higher probability than any other architectures. In this case, the Common Model had an exceedance probability of 96.4%, further confirming its superiority.

### 3.6. Effects of LFO Regressors

Unlike Di and Biswal's (2014) original study, in this analysis all the LFO regressors were left free to affect every region in the model equally. Although this could have, in principle, reduced the efficacy of intrinsic connectivity and altered the relative fit of the model, our results show that it did not. This raises the question of whether the eight LFO regressors were jointly needed, and, perhaps most interestingly, whether all frequencies do indeed affect all regions at rest. It is entirely possible, for example, that spontaneous brain activity arises in a subset of regions and propagates to others only through the whole-brain architecture; in Di and Biswal's (2014) paper, for example, low-frequency signals originate in the medial frontal cortex and propagate to the rest of the default-mode network from there.

**Figure 9:**
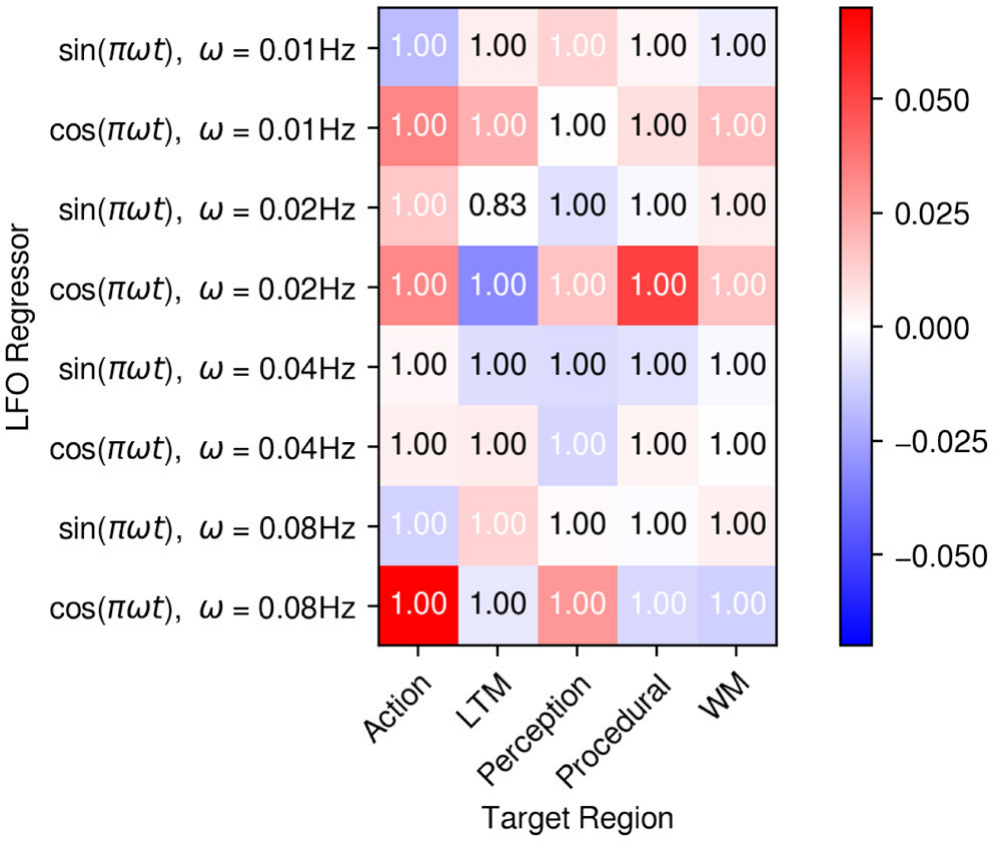
Mean average weights of the LFO drives on each region. The matrix visually depicts the mean estimated value of the matrix C in Equation (1). Colors represent the mean values, while the numbers on each cell represent the expected probability that each weight w is different than zero, *P*(|*w*|>0).

To test this, we examined the connectivity matrix **C** that defines the effects of regressors on regional activity. Individual parameter values for each entry in the matrix were averaged across participants using Bayesian Parameter Average, which takes into account the uncertainty associated with each individual parameter estimate. The resulting group-level averages are depicted in Figure 9, with the colors in each cell representing the mean weight value and the numbers that associated probability that such value is different than zero. All LFOs were found to have significant effects on all regions. Furthermore, different frequencies and phases turned out to have different effects on the five regions (Figure 9). This pattern of results is incompatible with the hypothesis that the LFOs are simply due to extrinsic, correlated physiological sources and suggests, instead, that LFOs might be intrinsic and specific to each region. In turn, this view is compatible both with the hypothesis that resting state represents generic priors and the view that LFOs might be jointly interacting with top-down processes to refine predictive models. To distinguish between these two hypotheses, the next section will examine the role of intrinsic connectivity.

### 3.7. Intrinsic Network Connectivity at Rest

Given that all LFO regressors had significant effects on all regions, it is important to examine the intrinsic connectivity parameters (matrix **A** in Equation 1). Although the likelihood analysis and the random effects analysis have yielded results similar to the previous findings (Stocco et al., 2021), the large amount of LFO drives could potentially explain most, if not all, of the variability in the BOLD signal at rest. Thus, it is important not only to see the relative fit of the model, but also whether all of the connectivity parameters between regions are significant and their values are comparable to those that were found in the original study.

**Figure 10:**
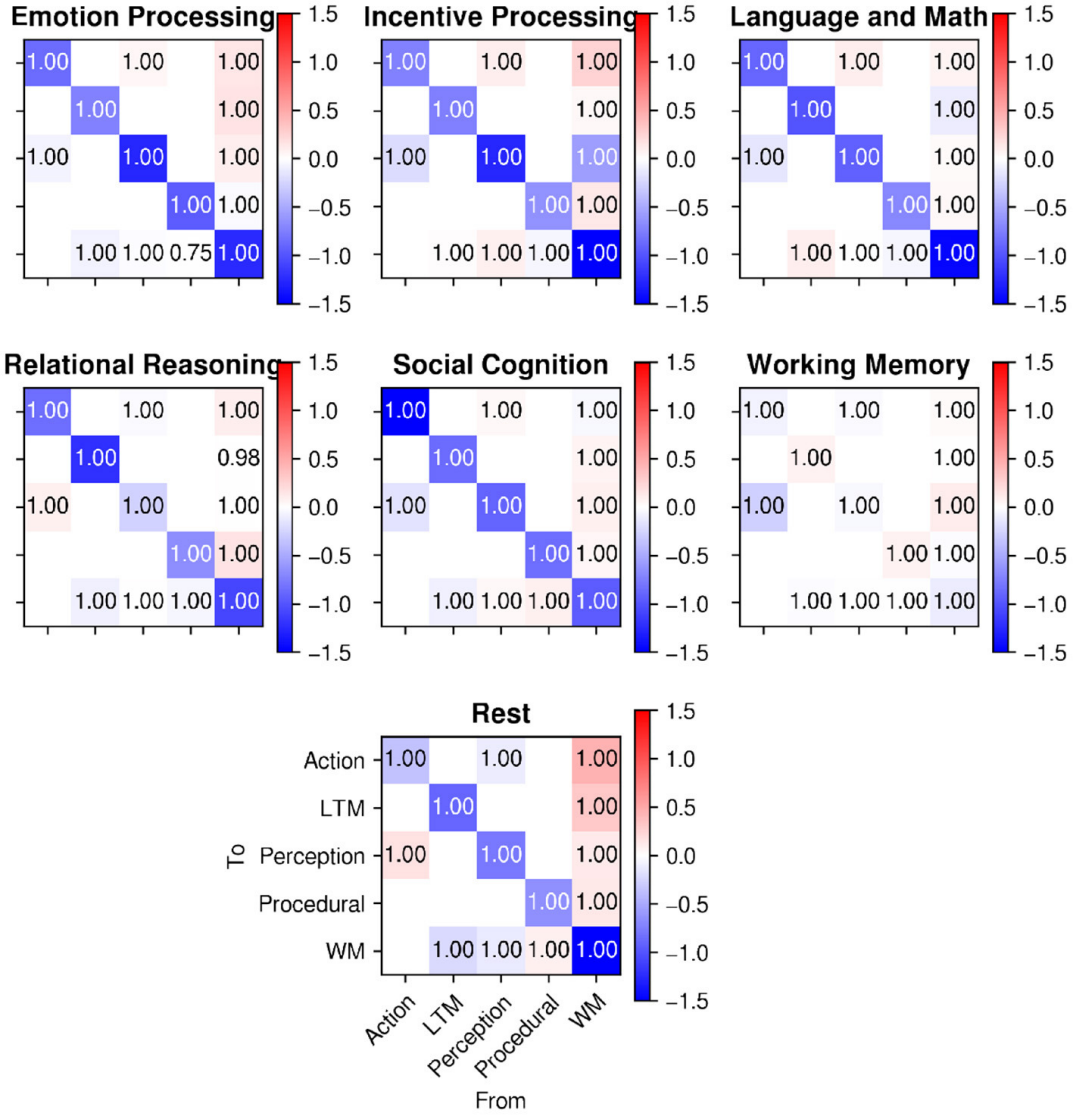
A comparison of intrinsic connectivity between regions (matrix A in Equation 1) during task-based activity (**top**; from Stocco et al., 2021) and during rest **(bottom)**. Cell colors represent connection weights; numbers in the cells represent posterior probabilities that the weights are different from zero.

Figure 10 shows the connectivity parameters (averaged across participants with the same Bayesian procedure that was used for matrix **C** in the previous section) for all of the HCP tasks used in Stocco et al. (2021), as well as the same parameters for the resting state analysis described here. As in Figure 9, colors in Figure 10 represent the estimated weights of each connection, while the numbers represent the probability that the weight is different from zero.

The data in Figure 10 shows that all of the connections between regions were significant, implying that the inter-region communication between regions along the network explained additional variance in the regional time-series that was not explained by the LFO regressors.

**Figure 11:**
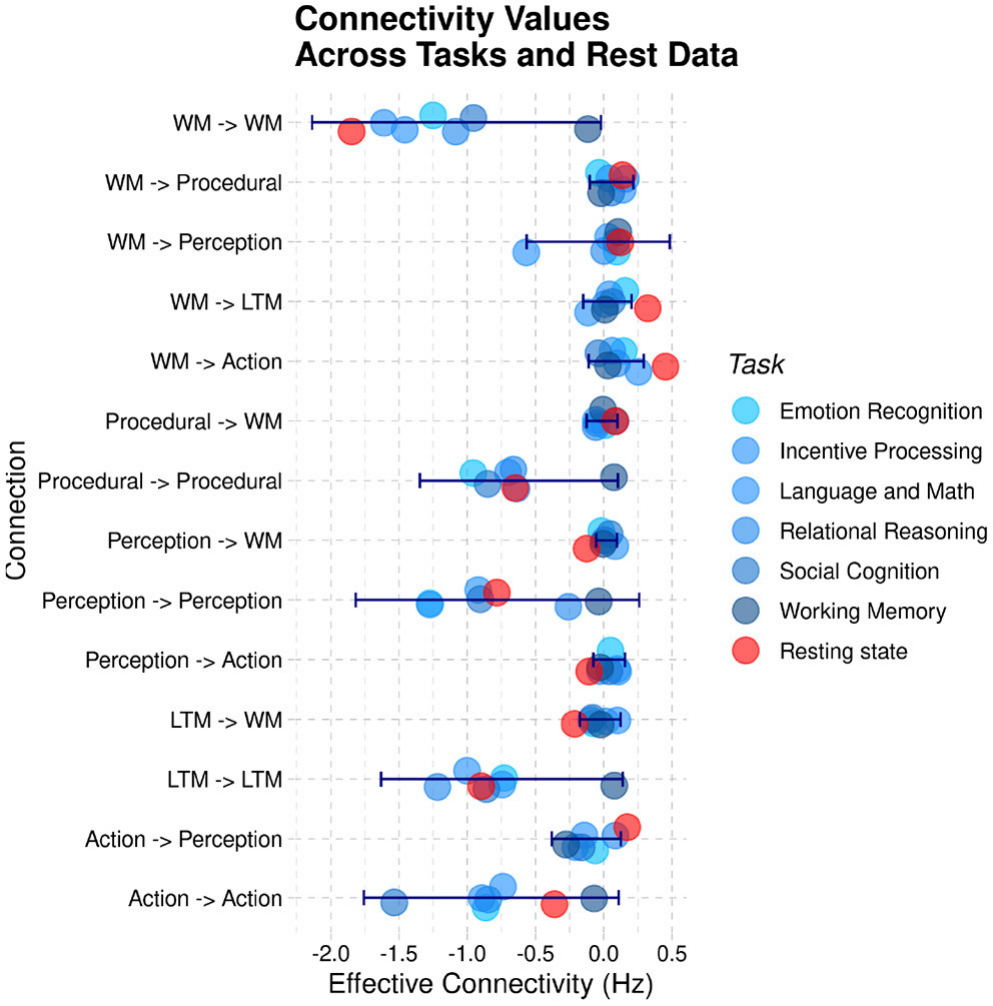
A comparison of the group-averaged intrinsic connectivity parameters derived from task-based recordings (blue) and resting-state sessions (red). The blue error bars represent the standard deviation of the task-based parameters.

In addition, the relative weights of the connections seem indistinguishable from those of the task-based data; specifically, the characteristic positive weights for the connectivity from the WM hub to the other regions is present in both the resting-state data and the task-based data. For ease of comparison, Figure 11 visualizes the different estimated values of the 14 connections across the six different HCP tasks of Stocco et al. (2021) (in different shades of blue) together with the corresponding resting-state parameter highlighted in red. A 2-by-14 ANOVA, with Paradigm (Task-based vs. Resting State) and Connection as factors, found an expected main effect of the Connection, [*F*_(1,13)_ = 15.413, *p* < 0.0001, highlighting the difference in strength in the directional connectivity between regions] but no main effect of Paradigm and no interaction (*F* < 0.95, *p*>0.51), confirming that the resting state parameter of the intrinsic connectivity did not differ significantly between task-based and resting-state recordings.

Although Figure 11 shows that all of the resting-state connectivity parameters were indeed within the range of variations of task-based parameters, it remains possible that their relationship was significantly different than what expected in during task. For example, one could expect that, compared to all task-based connectivity parameters, connectivity between perceptual regions and working memory is reduced in resting-state while connectivity between working memory and long-term memory is enhanced. To test whether this was the case, we calculated the mean Pearson correlation of the 14 connectivity parameters of the CMC architecture between all tasks and between all tasks and resting-state data. Because correlation coefficients are not normally distributed, mean values were computed by first transforming the Pearson correlation coefficients into *Z*-scores using Fisher's transform, then averaging the corresponding *Z*-scores, and eventually transforming the resulting mean *Z*-value back into a Pearson coefficient using the inverse transform. The mean correlation between all of the six task-based recordings was *r* = 0.84, ranging between 0.07 and 0.90. The mean correlation of resting state parameters with the task-based parameters was *r* = 0.83, almost identical to the expected correlations across tasks.

**Figure 12:**
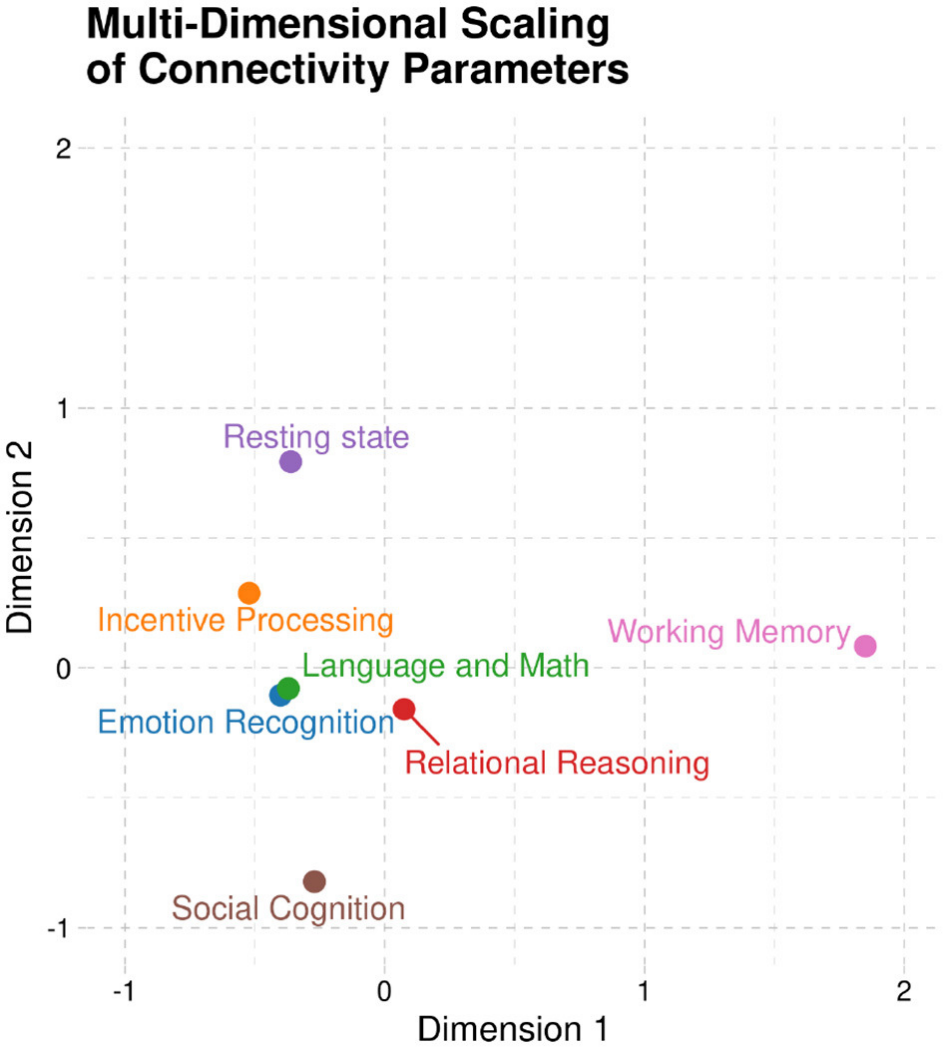
Multi-dimensional scaling of the correlations between effective connectivity parameters across all recording paradigms (six task-based and one resting-state). Note that Resting state does not significant stand apart from the task-based data, being, in fact, closer to the other tasks than the working memory paradigm.

Finally, to further visualize the degree of similarity between resting-state and task-based parameter, we carried out a multi-dimensional scaling (MDS) analysis, using the matrix of correlations between each paradigm as its similarity matrix. MDS results in a planar representation in which each paradigm is represented as a dot on a plane, and the axes of the plane are two abstract dimensions generated so that the Euclidean distances on the plan reflect the underlying similarity between recordings. The MDS results, shown in Figure 12, clearly illustrate that the resting state parameters do not differ significantly (that is, do not stand visually apart) from the task-based ones.

## 4. Discussion

The major finding of this paper is the apparent presence of an underlying structure of brain connectivity that accounts for activity even during undirected and task free behavior. The implications of these results are broad.

### 4.1. Implications for the Common Model of Cognition

The major finding of this paper is the apparent presence of an underlying structure of brain connectivity that accounts for activity even during undirected and task free behavior, and the similarity of that structure to the one that appears to underlie activity in tasks (Stocco et al., 2021). The CMC provides the best account of activity both in tasks and at rest, and this suggests that a single structure is responsible for the pattern of signals recorded in both states. The weights assigned to each network connection differ across tasks and between task and rest, indicating the flexible nature of a structure that adapts to the demands of a particular environment or task, but the configuration of connections appears to be consistent. The implications of these results are broad.

### 4.2. Implications for the Common Model of Cognition

First, these results demonstrate how the approach proposed by Stocco et al. (2021) to test general theories about the architecture of cognition through Dynamic Causal Modeling could be generalized to resting state through the use of simulated task events and meta-analytic ROIs, paving the way for future explorations of resting state data. In particular, the use of fMRI meta-analyses to determine ROIs presents the opportunity to explore increasingly complex model structures involving more specific brain areas. This approach to ROIs opens the door to examining each component in greater detail; separating visual perception from auditory perception, for example, or decomposing the long term memory component into semantic and episodic memory. The DCM framework also allows models to account for modulatory connections between regions, which, while not used in this paper, provide further opportunities to define and specify a general purpose framework of cognition.

While specific ROIs will always differ slightly across subjects and tasks, the meta-analytic ROIs used in the present study represent a much smaller search space than the broad parcellation used to define ROIs in the original CMC study. While the original masks in Stocco et al. (2021) were task-specific, the new ROI masks are optimized to include voxels that are most consistently active across a variety of different tasks tested in the literature. However, it should be acknowledged that the ROIs used in this analysis provide a very sparse map from which to locate the precise regions used in the modeling processes, and while they were selected to roughly correspond to the CMC's theoretical components, these are both very broad and in some cases incomplete. The Perception component, for example, incorporated only visual areas of the brain. This was done for the sake of simplicity, but future applications of this method of ROI generation should include additional sensory regions, either as part of a compound Perception ROI or as individual components. The application of the Nelder-Mead optimization also creates the possibility that the strongest signals originate from “contested” areas and are thus discounted, but serves the counter purpose of preventing spatially large clusters (like Perception and Action) from overwhelming signals from smaller clusters (like Procedural Memory). The advantage of combining meta-analytic initial masks from Neurosynth with a standardized method of optimization allows for future analyses based on networks of greater complexity than the CMC without the need to hand create region masks for each variation, but further exploration is required to judge the validity of this approach.

An additional weakness of this study lies in the simplicity of the model structure. The CMC provides the best account of underlying connectivity both in tasks and at rest, but it remains only the best model of those that we have tested so far, and is deliberately composed of a few, high level components. This top-down approach was partially constrained by the choice of using Dynamic Causal Modeling, which depends on a fundamental assumption of the brain's underlying architecture and its relevant connections and requires a predefined model structure, like the CMC, in order to make its predictions. As a test of convergent validity of the CMC, a data-driven approach was implemented (Hake et al., 2022) in an effort to derive potential network structure directly from brain activity. Granger Causality Modeling (GCM) of low-level functional brain activity was used to find causal connections between brain regions associated with the high-level cognitive components of the CMC. These new CMC model variations were then compared with the original CMC. The CMC was shown to have the greatest similarity to the network architecture uncovered by the GCM analysis, suggesting that the GCM architecture may be a variant of the CMC, as opposed to being intrinsically different. However, the possibility remains that the CMC is independently similar to a subset of task-specific networks and rest activity, rather than representing a single underlying framework used by both.

A third weakness of our approach is the small and limited number of components considered. Specifically, our architectures counted only five independent ROIs, corresponding to the five components of the CMC. While each ROI is large enough to encompass multiple, non-contiguous, bilateral brain regions (and is thus more similar to a functional network than a traditional ROI), large portions of the brain and many hypothetical components remain unmodeled. Notably, regions corresponding to the dorsal and ventral attentional networks in Power et al. (2011) and Yeo et al. (2011) and to the cingulo-opercular network in Power et al. (2011) are missing and their functional interpretation in terms of the CMC structure is difficult to establish. These conceptual difficulties might provide guidelines for the expansion and evolution of the CMC.

### 4.3. Implications for the Functional Role of Resting-State Activity

One of the goals behind this study was to test the applicability of large-scale architectures for task-based brain activity in a task-free paradigm, using only signals originating from spontaneous neural activity that would capture the intrinsic organization of the brain (Fox et al., 2005). The results suggest that, even at rest, the architecture that best accounts for the observed pattern of brain activity is the same one that was previously found to best fit task-based data across different domains (Stocco et al., 2021). This finding suggests a functional connection between task-based and resting-state activity. Furthermore, our findings suggest that spontaneous regional activity arises from the combination of region-specific oscillations and the general architecture that is used for task-based activity.

Taken together, these findings exclude some of the possible explanations for the functional role of resting-state activity. Specifically, the diversity of LFO effects across regions and the strong influence of the top-down cognitive architecture go against the hypothesis that spontaneous brain activity is due to either common noise or non-neural physiological sources, which would likely produce correlated LFOs across regions without a significant component of the common architecture. Instead, once the common architecture is accounted for, the residual oscillations that remain seem to be region-specific in terms of both phase and frequency.

In principle, the region-specific nature of LFOs is compatible with the hypothesis that spontaneous brain activity is used to maintain generic priors. The significant residual effect of intrinsic connectivity and the fact that it so strongly resembles the general architecture of task-based activity, however, go against it. Similarly, the fact that intrinsic connectivity so clearly resembles task-based effective connectivity suggests that resting-state activity is not a form of neural activity that is completely alternative to task-based activity, going against the alternate state hypothesis.

The only remaining hypothesis is that spontaneous brain activity is functionally related to the rehearsing and refining of generative, predictive activity (Pezzulo et al., 2021). This hypothesis is compatible with, and would indeed predict, our findings that spontaneous brain activity is best modeled as an interaction between a general-purpose network for task-based control and local low-frequency oscillation activity representing region-specific priors.

Our analysis, however, cannot pinpoint the specific mechanisms more precisely. In Pezzulo's conception, resting-state activity is produced by the spontaneous generation of signals by the set of brain networks responsible for top-down, predictive processing. While our results are certainly compatible with this view, they are also compatible with an alternative view, according to which spontaneous brain activity originates in the region-specific oscillations, and is then processed by the task-general cognitive architecture as a form of vicarious external stimuli. Future research will be needed to distinguish between these alternative views.

## Data Availability Statement

The raw data supporting the conclusions of this article will be made available by the authors, without undue reservation.

## Ethics Statement

Ethical review and approval was not required for the study on human participants in accordance with the local legislation and institutional requirements. The patients/participants provided their written informed consent to participate in this study.

## Author Contributions

CS: methodology, formal analysis, investigation, writing—original draft, writing—review and editing, and project administration. HH: methodology, investigation, visualization, and writing—review and editing. AS: conceptualization, methodology, formal analysis, resources, writing—original draft, visualization, supervision, project administration, and funding acquisition. All authors contributed to the article and approved the submitted version.

## Funding

This research was supported by grant FA9550-19-1-0299 from the Air Force Office of Scientific Research to AS.

## Conflict of Interest

The authors declare that the research was conducted in the absence of any commercial or financial relationships that could be construed as a potential conflict of interest.

## Publisher's Note

All claims expressed in this article are solely those of the authors and do not necessarily represent those of their affiliated organizations, or those of the publisher, the editors and the reviewers. Any product that may be evaluated in this article, or claim that may be made by its manufacturer, is not guaranteed or endorsed by the publisher.
